# Direct evidence of cheetah (*Acinonyx jubatus*) as intermediate host of *Toxoplasma gondii* through isolation of viable strains

**DOI:** 10.1186/s12917-024-03928-w

**Published:** 2024-02-24

**Authors:** Niuping Zhu, Hongjie Ren, Liulu Yang, Gaohui Mao, Junbao Li, Chunlei Su, Yurong Yang

**Affiliations:** 1 Veterinary Pathology, Henan Agricultural University, Zhengzhou, PR China; 2 Zhengzhou Zoo, Zhengzhou, PR China; 3 Department of Microbiology, University of Tennessee, Knoxville, USA

**Keywords:** *Toxoplasma gondii*, Cheetah (*Acinonyx jubatus*), Isolation, ToxoDB#319, ToxoDB#9, China, Genotyping

## Abstract

*Toxoplasma gondii* causes lifelong infection in most definitive and intermediate hosts. Clinical cases of toxoplasmosis in captive cheetahs have been reported. However, there are few reports of viable *T. gondii* strains isolated from cheetahs. Here, *T. gondii* infection was investigated using molecular and serological assays in cheetahs from China. Modified agglutination test (MAT) (cut-off: 1:25) indicated that all six examined cheetahs (*n* = 6) showed *T. gondii* antibodies. *Toxoplasma gondii* DNA was detected in three out of five cheetahs. Two viable *T. gondii* strains were isolated from the striated muscles of two cheetahs using mice bioassay. They were designated as TgCheetahCHn1 and TgCheetahCHn2. Genetic characterization of DNA derived from tachyzoites was performed using RFLP-PCR of 10 markers. *Toxoplasma gondii* TgCheetahCHn1 is ToxoDB PCR-RFLP genotype #319, and the alleles of ROP18/ROP5 types were 3/7. TgCheetahCHn2 is ToxoDB genotype #9, and the alleles of ROP18/ROP5 were 3/6. The average survival time of TgCheetahCHn1-infected Swiss mice was 22 ± 1 days (*n* = 23), and the mice did not have detectable *T. gondii*-specific antibodies until 117 ± 30 days post-inoculation (*n* = 8), therefore, TgCheetahCHn1 had intermediate virulence. TgCheetahCHn2 was avirulent for Swiss mice. Few brain tissue cysts (0–50) were observed in the mice inoculated with TgCheetahCHn1 or TgCheetahCHn2. The results provide direct evidence of cheetah as intermediate host of *T. gondii*.

**Supplementary Information:**

The online version contains supplementary material available at 10.1186/s12917-024-03928-w.

## Background

*Toxoplasma gondii* causes lifelong infection in most definitive and intermediate hosts. Felids, including cheetahs (*Acinonyx jubatus*), are the definitive host in the epidemiology of toxoplasmosis, they can excrete environmentally resistant oocysts [1, 2]. Felids become infected by ingesting meat containing cysts, water or food contaminated with oocysts [3–5].

Cheetahs are listed as a vulnerable species, and only 6,517 mature cheetahs currently exist [6]. Studies have reported serological responds to *T. gondii* in captive and wild cheetahs [7–9] and have found *T. gondii* infection in cheetahs using molecular or histological methods [10, 11]. However, there is no viable *T. gondii* strain isolated from cheetahs. Moreover, there is no previous survey of cheetahs infected with *T. gondii* in China. In fact, these cheetahs in China were imported from Africa. Due to the lack of *T. gondii* testing at the time of importation, it is difficult to ascertain the exact timing of infection. In the present study, *T. gondii* infection in cheetahs was identified using molecular and serological assays, and bioassays in mice.

## Materials and methods

### Samples collection and informations

Between 2017 and 2021, five dead (3 dead of chronic renal insufficiency, 2 dead of natural aging) cheetahs (*Acinonyx jubatus*) and one cheetah uterus and serum sample from zoos in Henan Province (China) were examined for *T. gondii* (Table 1). Tissue samples including the heart, leg muscles, kidneys, spleen, lungs, liver, and intestines from all five dead cheetahs were collected. In addition, lymph nodes, and tongues from cases 3, 5, and 6 were also collected. The fresh tissue samples were stored at 4℃ and sent to the Lab of Veterinary Pathology, Henan Agricultural University for pathological diagnosis.

**Table 1:** Background and isolation of *Toxoplasma gondii* from cheetahs

Samples	Samples received date	Age (in years), sex	Clinical symptoms	Autopsy and pathological findings	Formalin fixed tissues	Toxoplasma gondii PCR-positive tissue	MAT	Mice bioassay^c^	
								Swiss	IFN-γ^−/−^
Case 1^a^	Apr 20, 2017	≥ 13, female	Anorexia; discharge of dark red thick fluid from the vagina. After the surgical removal of the uterus, the cheetah is alive.	Acute catarrhal endometritis.	Uterus	nd	≥ 1:200^b^	nd	nd
Case 2	May 22, 2017	17, male	Shortness of breath; limb spasm; 39.4 °C; and died the next day.	Pulmonary congestion; spleen atrophy; and abundant lipofuscin deposition in hepatocytes.	Heart, liver, spleen, lungs, kidneys, intestines	Striated muscle digestive fluid	≥ 1:200^b^	0/5	nd
Case 3 (TgCheetahCHn1)	Dec 20, 2020	4, male	Weight loss; vomiting; and trembling. Renal failure (confirmed by biochemical tests), no obvious improvement after therapy, and then died.	Dark yellow liver; pulmonary emphysema; glomerulus atrophy; septic spleen; hepatocyte atrophy; and intestinal necrosis.	Heart, liver, spleen, lungs, kidneys, intestines, tongue, diaphragm, leg muscles, lymph nodes	Myocardium, liver, spleen, lung, diaphragm, tongue, leg muscle, and striated muscle digestive fluid	1:25	2/2	2/2
Case 4 (TgCheetahCHn2)	Jan 31, 2021	21, male	Anorexia for 10 days, no improvement after antibiotic treatment, and then died.	Pulmonary congestion; gray-green renal pelvis; unclear boundary between the cortex and medulla; interstitial pneumonia; atrophy of spleen, liver, and kidney; renal failure; and atherosclerosis.	Heart, liver, spleen, lungs, kidneys, skeletal muscles, pancreas	Myocardium, liver, spleen, lung, and striated muscle digestive fluid	1:400	5/5	2/2
Case 5	Apr 24, 2021	Adult, male	No obvious abnormal symptoms and died suddenly.	Hepatomegaly; multifocal yellow-gray nodules in the spleen; intracardial hemorrhage; swollen kidney; congested medulla; pulmonary congestion; acute interstitial pneumonitis; necrotic splenitis; and chronic renal insufficiency.	Heart, liver, spleen, lungs, kidneys, small intestines, tongue, diaphragm, skeletal muscles, lymph nodes	Negative	1:100	0/5	nd
Case 6	Dec 30, 2021	5, male	Dry nose, dehydration, and then died.	Congestive hepatomegaly; endocardial hemorrhage; hard and swollen kidney; chronic atrophic glomerulonephritis; and septic spleen.	Heart, liver, spleen, lungs, kidneys, intestines, tongue, lymph nodes, pancreas	Negative	1:50	0/4	0/1

^a^ Only uterus and blood samples were collected

^b^ End titration not performed

^c^ Number of positive mice/numbers of inoculated mice

nd: Experiment not done

### Mice

Swiss mice were supplied by Zhengzhou University Laboratory Animal Center (Zhengzhou, China). IFN-γ^−/−^ mice were purchased from Jackson laboratory (Stock No. 002287; Bar Harbor, ME, USA) and then bred in house. The animal use protocol was approved by the Beijing Association for Science and Technology (Approval SYXK [Beijing] 2007-0023).

### Detection of antibodies against *T. gondii* in cheetah heart juice or serum

Heart juice or serum samples from the cheetahs (*n* = 6) were tested for *T. gondii* antibodies using the modified agglutination test (MAT) [12]. The MAT antigen of formalin-treated *T. gondii* was obtained from the University of Tennessee Research Foundation (Knoxville, TN, USA). *Toxoplasma gondii*-positive (VEG infected mouse serum) and *T. gondii*-negative (Specific Pathogen Free) mice sera were provided by Dr. J. P. Dubey (Beltsville, ARS, USDA, USA). In cases 1 and 2, serum and heart juice samples were tested at titers of 25 and 200. In cases 3, 4, 5 and 6, heart juice samples were tested at titer of 25, and the titer was then doubled to 3,200. Negative and positive controls were included in each plate.

### Histopathological analysis

Tissue from the cheetahs were formalin fixed (Table 1), paraffin embedded, sectioned, and stained with H&E. The cheetah tissues suspected infected with *T. gondii* was performed immunohistochemical (IHC) staining [13]. The primary antibody was rabbit anti-*T. gondii* polyclonal antibody, the second antibody was anti-rabbit specific antibody from Abcam (USA, ab64264).

### Isolation *T. gondii* from cheetah tissues by bioassay

Tissue samples from five cheetahs were bioassayed in mice [3] (Table 1). Tissue samples (50 g; namely heart, leg muscle, tongue, diaphragm, or brain) from the cheetahs were pepsin digested and inoculated into Swiss mice (*n* = 2–5) or IFN-γ^−/−^ mice (*n* = 2). The mice were checked daily. It was two batch mice for TgCheetahCHn1. The first batch mice were documented the survival time without medicine intervention, then make a statistical analysis. The second batch mice was given sulfadiazine in water after 2 weeks inoculation TgCheetahCHn1. Mice blood or heart fluids were collected after 30, 60-, 90-, 120-, and 155-days post-inoculation (DPI) or natural dead mice. The antibody titer was checked at 31, 61, 91,121, and 155 DPI. It was one batch mice for TgCheetahCHn2, mice blood was collected after 30, and 60-DPI. In brief, about 0.3 ml blood from mice facial vein was collected and centrifuged for 10 min at 10,000 rpm, and serum *T. gondii* antibodies were tested using MAT. Lungs or brains of the natural dead mice during the experiment were examined for *T. gondii* tachyzoites or tissue cysts, as appropriate. If parasites were not found in mice tissues, homogenized lung, brain, and heart tissues were subcutaneously subpassaged into a new group of mice. PCR and IHC staining were performed on mice suspected to be infected with *T. gondii*. The criteria used to suspect mice toxoplasmosis include depress, decreased appetite, pulmonary congestion, and markedly enlarged spleen and mesenteric lymph nodes after inoculated the parasites.

*Toxoplasma gondii* negative mice were subpassaged one time only, positive mice tissues were seed into Vero cell (provide by Shanghai yiyan bio-technology Co. Ltd., China) and cryopreservation in liquid nitrogen.

### Extraction and detection of *T. gondii* DNA by PCR in cheetahs’ tissues

*Toxoplasma gondii* DNA was tested in the frozen tissues and pepsin-digested tissues from the cheetahs using PCR assays [14]. The DNA from the tissues and pepsin digested samples was extracted using a commercial DNA extraction kit (Tiangen Biotec Company, China, DP 304). Briefly, 30 mg tissues was treated with sodium dodecyl sulfate/proteinase K at 56℃ for 12 h digestion. DNA samples were purified by silica gel column chromatography and eluted into 50 µl elution buffer. For *T. gondii*, it was 25 µl in total, with 12.5 µl added into 2×PCR Mix (Kangrun Biological Co., LTD, China), 1 µl each primer (Shenggong Bioengineering Co., LTD, Shanghai, China), 2 µl of the DNA sample, and the remaining volume made up with ddH_2_O (Kangrun Biological Co., LTD, China). Mixed and then put into PCR instrument (T100, BIO-RAD) for amplification. The *T. gondii* amplification program was initial denaturation at 94 °C for 2 min; 35 cycles of amplification (94 °C for 1 min, 60 °C for 1 min, and 72 °C for 1 min) and final extension at 72 °C for 10 min, 6 °C forever. *Toxoplasma gondii* were detected using the specific primer pairs TOX5/TOX8 (5′-CGCTGCAGACACAGTGCATCTGGATT-3′ and 5′-CCCAGCTGCGTCTGTCGGGAT-3′) by PCR [14]. Negative PCR controls and the positive control were included in all batches. The *T. gondii* PCR products were expected 450 bp in length.

### Cell cultivation

In order to obtain *T. gondii* tachyzoites for genotyping and assess the virulence by inoculating mice. *T. gondii*-infected mice tissues homogenates (lung, mesenteric lymph node, and brain) were seeded into Vero cell culture flasks as previously described [3].

### Genotyping

The DNA from tachyzoites was extrated from comercial kit., The extraction method for tachyzoites DNA was same as for tissues DNA. The genotype of isolated *T. gondii* strains was checked by multiplex PCR using 10 PCR-RFLP genetic markers [15]. The virulence-predictive genes ROP5 and ROP18 were also typed [16]. And the reference *T. gondii* DNA (GT1, PTG, CTG, MAS, TgCgCa1, TgCatBr5, TgCatBr64 and TgRsCr1) was included in each batch.

### Evaluation the virulence of *T. gondii* TgCheetahCHn1 and TgCheetahCHn2 strains

The virulence of *T. gondii* strains isolated from the cheetahs was evaluated in Swiss mice [17]. TgCheetahCHn1 and TgCheetahCHn2 *T. gondii* tachyzoites were counted and diluted 10-fold from 10^4^ to reach an endpoint of < 1 tachyzoite. Next, the tachyzoites were inoculated into five mice each per dilution intraperitoneally, and the clinical symptoms were checked daily. For TgCheetahCHn2, on 30, 60- DPI, the mice were bled and tested for antibodies to *T. gondii* using MAT, with titers 25 and 200. For TgCheetahCHn1, blood were collected from the survival mice after 30, 60-, 90-, and 120-DPI, and 25 and 200 titers were tested for *T. gondii* antibodies Then, the mice were euthanized on 120 DPI (euthanasia methods: 0.3% pentobarbital, 0.2 mL per mice first, then 10% potassium chloride, 0.1 mL per mice, intraperitoneally), their brains cysts were examined and enumerated. All the mice tissues were fixed in formalin. The *T. gondii* virulence was evaluated according to the percentage of dead mice among *T. gondii*-positive mice.

### Experiment information of statistical analysis with *T. gondii* strains isolated from cheetahs

GraphPad Prism 8.0 software was used statistical analyses. The data were analyzed using the chi-square test. The values are expressed as mean ± SEM, and *P* < 0.05 is considered significant.

## Results

### Histopathological findings and *T. gondii* infection

*Toxoplasma gondii* parasites were not found in the IHC and H&E-stained tissue sections of the six cheetahs. Chronic renal insufficiency (4/5), interstitial pneumonia (2/5), necrotic splenitis (1/5), splenatrophy (2/5), septic spleen (1/5), systemic atrophy (1/5), arterial sclerosis (1/5), and acute endometritis (1/1) were observed through microscopic examinations. The findings showed that none of the cheetahs died of acute toxoplasmosis (Table 1).

### Detection of *T. gondii* antibodies and DNA

The MAT indicated that all six examined cheetahs showed *T. gondii* IgG antibody, with titers of 25 in one case, 50 in one case, 100 in one case, 400 in one case, and ≥ 200 in two cases. *Toxoplasma gondii* DNA was detected in three out of five cheetahs. *Toxoplasma gondii* DNA was found in case 2 (pepsin digested striated muscle), case 3 (myocardium, spleen, liver, lungs, diaphragm, tongue, skeletal muscle, and pepsin digested striated muscle), and case 4 (myocardium, liver, spleen, lungs, and pepsin digested striated muscle) (Table 1).

### Isolation of *T. gondii* strains and their genetic characterization

**Fig. 1:**
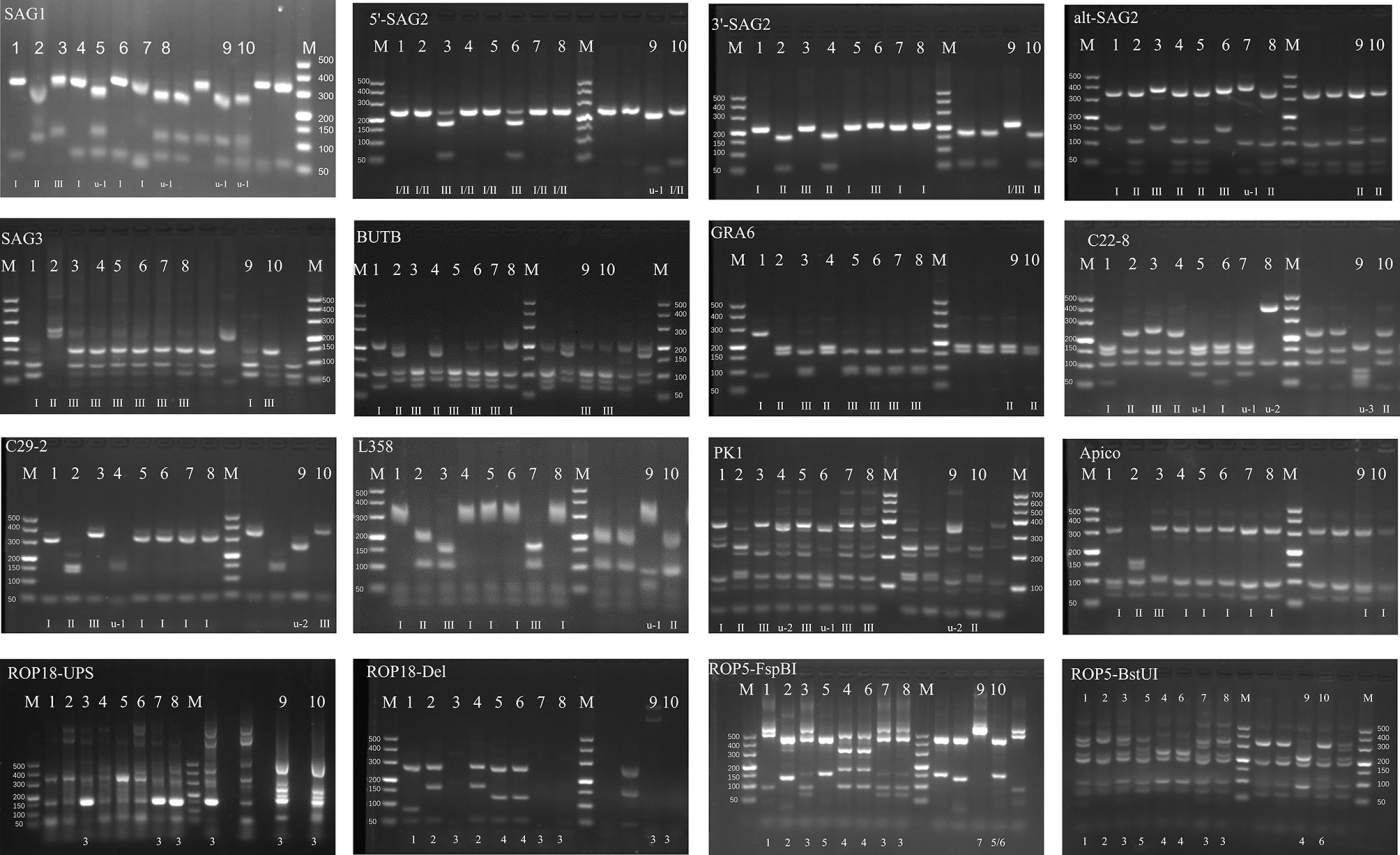
Genotyping of *T. gondii* TgCheetahCHn1 and TgCheetahCHn2 from cell culture. 1:GT1, 2:PTG, 3:CTG, 4:TgCgCal, 5:MAS, 6:TgCatBr5, 7:TgCatBr64, 8:TgToucan (TgRsCr1), 9:TgCheetahCHn1, 10:TgCheetahCHn2, M:markers

The tissues of five cheetahs were bioassayed in mice individually (Table 1). For cheetah case 3, the Tox#21 − 3 mice group (two Swiss mice: M#521 and M#540 and two IFN-γ^−/−^ mice: M#518 and M#539), M#521 had *T. gondii* antibodies on 30 DPI with a titer of 1:25, and 50 cysts were recovered from the brain on 37 DPI. The IFN-γ^−/−^ mice (M#518 and M#539) died on 27 DPI and 28 DPI due to toxoplasmosis, and tachyzoites were found in the lungs. However, this *T. gondii* strain (TgCheetahCHn1) from the tissues of the mice (lungs, brain, lymph nodes, or spleen) was difficult to propagate in Vero cell cultures until subpassaged six times in Swiss mice. Furthermore, the average survival time of TgCheetahCHn1-infected Swiss mice (without medicine intervention) was 22 ± 1 days (*n* = 23). For survivor (given sulfadiazine after 2 weeks inoculation), the mice did not have detectable *T. gondii*-specific antibodies until 117 ± 30 DPI (*n* = 8) (Supplementary Table 1). The genotype of TgCheetahCHn1 was a mixed type of I, II, and III, and it belonged to ToxoDB genotype#319, which was determined using PCR-RFLP with 10 markers. In addition, the ROP18 and ROP5 gene allele types of this isolate were 3/7 (Fig. 1; Table 2).

**Table 2:** Genotypes of *Toxoplasma gondii* isolates from cheetahs according to PCR-RFLP of 10 markers and virulence proteins

Isolated ID	SAG1	(3′+5′) SAG2	Alt SAG2	SAG3	BTUB	GRA6	C22-8	C29-2	L358	PK1	Apico	Genotype ToxoDB	ROP18	ROP5
GT1, reference	I	I	I	I	I	I	I	I	I	I	I	#10	1	1
PTG, reference	II	II	II	II	II	II	II	II	II	II	II	#1	2	2
CTG, reference	II/III	III	III	III	III	III	III	III	III	III	III	#2	3	3
TgCgCa1, reference	I	II	II	III	II	II	II	u-1	I	u-2	I	#66	2	5
MAS, reference	u-1	I	II	III	III	III	u-1	I	I	III	I	#17	4	4
TgCatBr5, reference	I	III	III	III	III	III	I	I	I	u-1	I	#19	4	4
TgCatBr64, reference	I	I	u-1	III	III	III	u-1	I	III	III	I	#111	3	3
TgRsCr1, reference	u-1	I	II	III	I	III	u-2	I	I	III	I	#52	3	3
TgCheetahCHn1	u-1	u-1	II	I	III	II	u-3	u-2	u-1	u-2	I	#319	3	7
TgCheetahCHn2	u-1	II	II	III	III	II	II	III	II	II	I	#9	3	6

For cheetah case 4, in the Tox#21 − 7 mice group (five Swiss mice: M#827, 832, 859, 860, and 861). A few cysts (*n* = 20) were found in the brain M#832 (58 DPI) and M#861 (99 DPI). The *T. gondii* strain from the tissues of M#832 and M#827 (Tox#21 − 7) was propagated in cell cultures on 20 DPI and designated TgCheetahCHn2. The genotype of TgCheetahCHn2 belonged to ToxoDB genotype#9 (*Chinese1*), and the ROP18 and ROP5 gene allele types of this isolate were 3/6 (Fig. 1; Table 2). Overall, the Swiss mice did not have detectable *T. gondii*-specific antibodies for TgCheetahCHn2 until 50 ± 11 DPI (*n* = 15) (Supplementary Table 1).

For the other three cheetah cases (cases 2, 5, and 6), none of the mice (*n* = 5) had *T. gondii* antibodies, and no tissue cysts were observed on 60 DPI.

### Evaluation of the virulence of the *T. gondii* TgCheetahCHn1 and TgCheetahCHn2 strains using mice

**Fig. 2:**
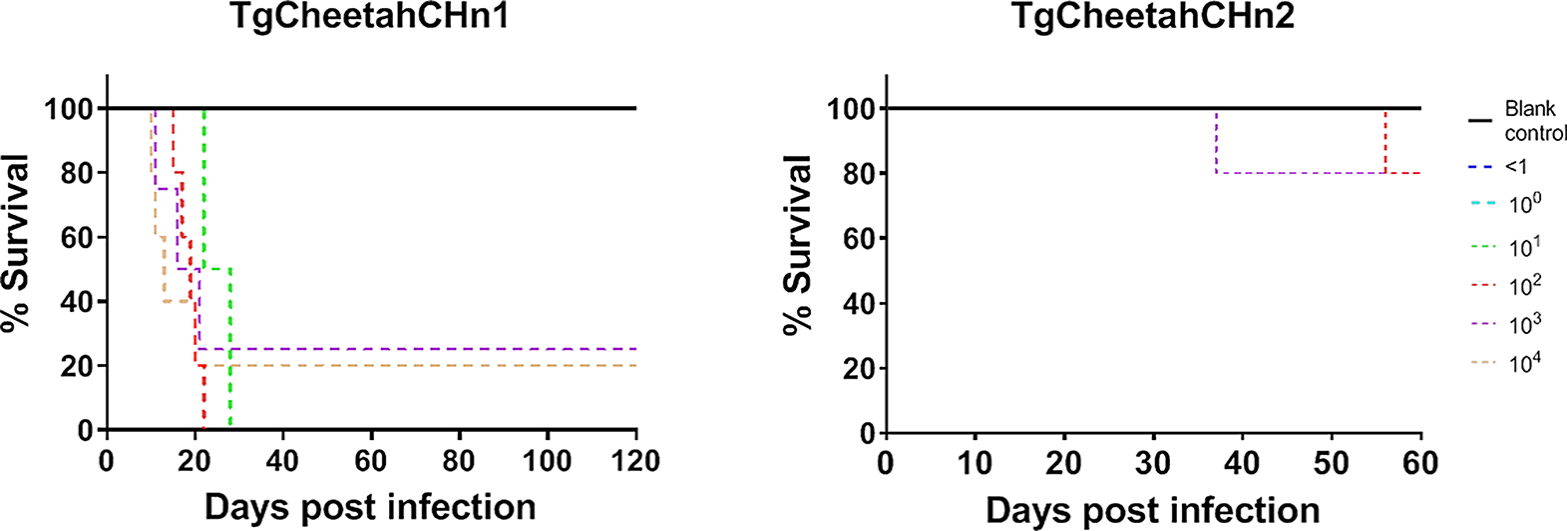
Swiss mice survival curves after infected *T. gondii* isolated strains from cheetahs. Survival curves were generated using the GraphPad Prism 8.0. For TgCheetahCHn1, none of the control mice and inoculation < 1 doses of *T. gondii* tachyzoites died, whereas the survival rate of mice inoculated with 1–10^4^ doses of tachyzoites was 100% (*P* > 0.05), 0% (*P* < 0.05), 0% (*P* < 0.05), 25% (*P* < 0.05), and 20% (*P* < 0.05), for Swiss mice, respectively. For TgCheetahCHn2, none of the control mice and inoculation < 1 doses of *T. gondii* tachyzoites died, whereas the survival rate of mice inoculated with 1–10^4^ doses of tachyzoites was 100%, 100%, 80%, 80%, and 100%, for Swiss mice, respectively (*P* > 0.05)

After inoculating mice with different doses of TgCheetahCHn1 tachyzoites, most *T. gondii-*infected mice survival time (82%, 14/17) was less than 30 days due to acute toxoplasmosis (Fig. 2). A dose of 100 tachyzoites was infective for all the inoculated mice (detectable *T. gondii*-specific antibodies or *T. gondii* DNA). However, none of the *T. gondii* cysts were found in the mice brains.

After inoculating mice with TgCheetahCHn2 tachyzoites, most *T. gondii-*infected mice survival time (92%, 23/25) was more than 60 days (Fig. 2). A dose of one tachyzoite was infective for the mouse. In the 10^2^ and 10^4^ tachyzoite groups, only a few *T. gondii* cysts (2–10) were observed in the mice brains when euthanized on 61 DPI.

## Discussion

In the present study, *T. gondii* infection was investigated in six captive cheetahs from zoos in China. The MAT revealed that all the cheetahs had *T. gondii*-specific antibodies (cut-off: 1:25). And high prevalence of *T. gondii* infection in captive cheetahs has been reported in several studies [1, 7, 8]. The validity of MAT was supported by the isolation of *T. gondii* from lambs, pigs, and chickens [18–20]. However, our understanding of the specificity of MAT in cheetahs is still limited. In this study, *T. gondii* strains were successfully isolated from case 3 cheetah tissues with a MAT titer of 25 and case 4 cheetah tissues with titer of 400. This result suggests that a MAT titer of 25 might indicate *T. gondii* infection in cheetahs.

Researches about the seroprevalence of *T. gondii* in cheetahs are limited worldwide [7–9]. Captive cheetahs have a higher seropositivity rate of *T. gondii* than free-range cheetahs, possibly because captive cheetahs live in zoos under environmental stress. In this study, the clinical and pathological diagnosis show that the cheetahs were died of aging or renal insufficiency. None of the six cheetahs produced any offspring. Compared to wild cheetahs, the reproductive problems and renal insufficiency of captive cheetahs may also be related to environmental stress [21].

In present study, *T. gondii* DNA was found in cases 2, 3, and 4, but viable *T. gondii* strains were only isolated from cases 3 and 4 (Table 1). However, for all the cheetah cases, no *T. gondii* tissue cyst or tachyzoite was found in tissue sections. It may be due to the relatively low density of *T. gondii* parasites in the tissues. These results showed that the serological, molecular, and bioassay methods were more sensitive than histological methods for diagnosis of *T. gondii* infection in these cheetahs. This finding is agree with researches on other animals [13, 18, 22, 23]. Previously, *T. gondii* parasites were observed in the heart, liver, brain, lung, pancreas, spleen, intestinal mucosa, and urinary bladder epithelium in captive cheetahs with acute toxoplasmosis [10, 11].

The main infection route for cheetahs might be eat meat containing *T. gondii* bradyzoites or oocysts from water and the environment. Our results indicate that cheetahs from central China are exposed to *T. gondii* widely. Their food includes raw beef and pork. The seroprevalence of *T. gondii* infection in cattle and swine from China is 9% and 33%, respectively [24]. Zookeepers may be an important factor for transmission of *T. gondii* oocysts [25].

In this study, the virulence of two *T. gondii* strains was evaluated in mice. The results indicate that TgCheetahCHn1 has intermediate virulence to mice (82% died within 30 DPI of acute toxoplasmosis), but the tachyzoites were very few in the lungs or mesenteric lymph nodes, and they grew very slowly in vitro. Furthermore, if the mice survived, TgCheetahCHn1 induced a delayed humoral immunological response (117 ± 30 DPI). The TgCheetahCHn2 strain was avirulent to mice. The performance of *T. gondii* infection vary with the strain of the parasite, the host-species, the host immune status and the infective dose or the stage of the parasite [3, 26].

This study is the first report of *T. gondii* isolated from cheetahs. The *T. gondii* TgCheetahCHn1 strain belongs to ToxoDB#319. ToxoDB#319 may be a new mutant strain, with an atypical u-3 allele at the c22-8 locus. The TgCheetahCHn2 strain belongs to ToxoDB#9. Genotype #9 (*Chinese1*), #2, #4, #20, #17, #292, #1, #204, #205, and #10 have identified in animals from China; ToxoDB #9 and #2 are the endemic genotypes in China [27–33].

Although TgCheetahCHn1 had intermediate virulence to mice, the asexual reproduction stage of this strain was slow, both in vivo and in vitro. Thus, the genes responsible for the phenotypic differences need to be explored in the future. It was demonstrated that genetic background of ROP18 and ROP5 genes are associated with mouse-virulence of *T. gondii* [15]. The allele type of ROP18/ROP5 for TgCheetahCHn1 was 3/7. TgCheetahCHn2 (genotype #9) was an avirulent phenotype, with 3/6 of ROP18/ROP5 gene type. However, there was no other report about virulence of these two ROP18/ROP5 gene types, except for *T. gondii* TgMonkeyCHn1 with 3/6 (avirulent) [13].

*Toxoplasma gondii* antibodies in mice (infected with cysts orally) can be detected on 12 DPI [34]. Generally, IgG can cooperate with macrophages to resist the secondary invasion of *T. gondii*. The antiparasitic function of IgG antibodies is mainly achieved by inhibiting the adhesion of parasite and host cell receptors [35, 36]. However, *T. gondii* releases many factors to disrupt this exclusion mechanism and establish infection [37]. TgCheetahCHn1 induced delayed humoral immunological response in mice. The difference between *T. gondii* TgCheetahCHn1 and other strains will give a reference for vaccine research.

The cheetah is proven definitive host of *T. gondii* by bioassays of fecal samples in mice [2]. In this study, two viable strains were isolated from two cheetahs, and they might have shed oocysts in the feces. In addition, the findings provide direct evidence of cheetah as intermediate host of *T. gondii*. An effective way to reduce the risk of *T. gondii* exposure for felids would be to freeze meat thoroughly before feeding. Further, inactivating the oocysts from felids by burning or incubating at high temperatures are important strategies for controlling *T. gondii* transmission.

## Conclusion

MAT titer of 25 might indicate *T. gondii* infection in cheetahs. The *T. gondii* TgCheetahCHn1 strain belongs to ToxoDB#319. The TgCheetahCHn2 strain belongs to ToxoDB#9 and is the endemic genotype found in China. ToxoDB#319 may be a new mutant strain, with an atypical u-3 allele at the c22-8 locus. TgCheetahCHn1 had intermediate virulence, and TgCheetahCHn2 was avirulent for Swiss mice. This study provides evidence of cheetahs as intermediate hosts of *T. gondii*. Cheetahs in zoos may indirectly or direct transmit *T. gondii* to other animals and humans.

### Electronic supplementary material

Below is the link to the electronic supplementary material.

Supplementary Material 1

Supplementary Material 2

## Data Availability

The data supporting the findings of this study are available from the corresponding author upon request. The TgCheetahCHn1 or TgCheetahCHn2 isolates were cryopreserved and available for the further analysis.
