# Supplementary material for: Direct evidence of cheetah (Acinonyx jubatus) as intermediate host of Toxoplasma gondii through isolation of viable strains
Source: BMC Vet Res. 2024 Feb 24;20:71. doi: 10.1186/s12917-024-03928-w (PMC10893619; doi:10.1186/s12917-024-03928-w)
Supplement: Supplementary file 1 — Supplementary Material 1 [file 12917_2024_3928_MOESM1_ESM.pptx]

## Slide 1
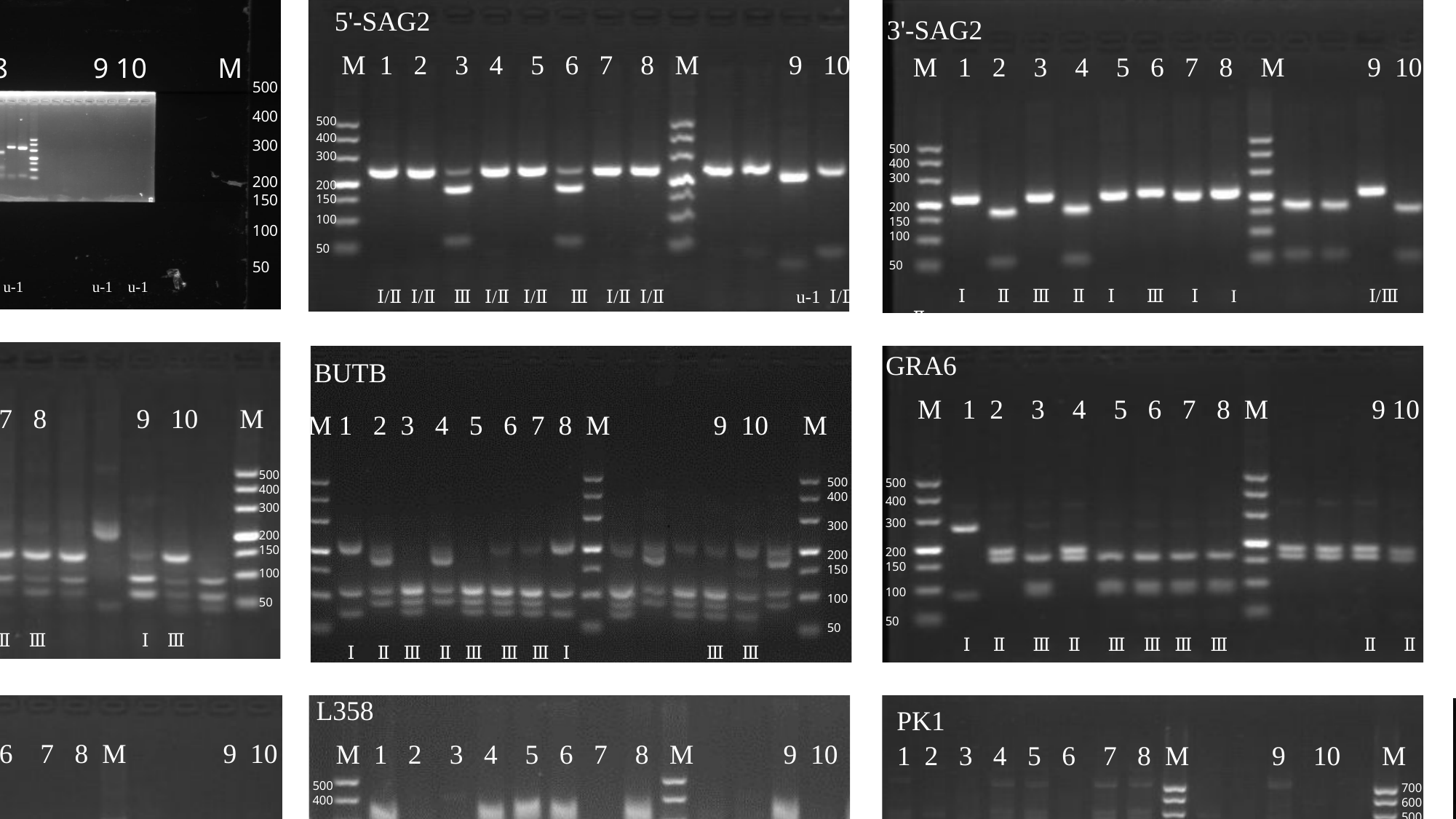

SAG1
500
400
300
200
150
100
50
 1 2 3 4 5 6 7 8 9 10 M
 Ⅰ Ⅱ Ⅲ Ⅰ u-1 Ⅰ Ⅰ u-1 u-1 u-1
M 1 2 3 4 5 6 7 8 M 9 10
 Ⅰ/Ⅱ Ⅰ/Ⅱ Ⅲ Ⅰ/Ⅱ Ⅰ/Ⅱ Ⅲ Ⅰ/Ⅱ Ⅰ/Ⅱ u-1 Ⅰ/Ⅱ
500
400
300
200
150
100
50
5'-SAG2
alt-SAG2
500
400
300
200
150
100
50
 Ⅰ Ⅱ Ⅲ Ⅱ Ⅱ Ⅲ u-1 Ⅱ Ⅱ Ⅱ
 M 1 2 3 4 5 6 7 8 M 9 10
3'-SAG2
500
400
300
200
150
100
50
 Ⅰ Ⅱ Ⅲ Ⅱ Ⅰ Ⅲ Ⅰ I Ⅰ/Ⅲ Ⅱ
 M 1 2 3 4 5 6 7 8 M 9 10
SAG3
500
400
300
200
150
100
50
 Ⅰ Ⅱ Ⅲ Ⅲ Ⅲ Ⅲ Ⅲ Ⅲ Ⅰ Ⅲ
M 1 2 3 4 5 6 7 8 9 10 M
GRA6
500
400
300
200
150
100
50
 Ⅰ Ⅱ Ⅲ Ⅱ Ⅲ Ⅲ Ⅲ Ⅲ Ⅱ Ⅱ
 M 1 2 3 4 5 6 7 8 M 9 10
BUTB
500
400
300
200
150
100
50
 Ⅰ Ⅱ Ⅲ Ⅱ Ⅲ Ⅲ Ⅲ Ⅰ Ⅲ Ⅲ
M 1 2 3 4 5 6 7 8 M 9 10 M
C22-8
500
400
300
200
150
100
50
 Ⅰ Ⅱ Ⅲ Ⅱ u-1 Ⅰ u-1 u-2 u-3 Ⅱ
 M 1 2 3 4 5 6 7 8 M 9 10
C29-2
500
400
300
200
150
100
50
 Ⅰ Ⅱ Ⅲ u-1 Ⅰ Ⅰ Ⅰ Ⅰ u-2 Ⅲ
 M 1 2 3 4 5 6 7 8 M 9 10
L358
500
400
300
200
150
100
50
 Ⅰ Ⅱ Ⅲ Ⅰ Ⅰ Ⅰ Ⅲ Ⅰ u-1 Ⅱ
 M 1 2 3 4 5 6 7 8 M 9 10
700
600
500
400
300
200
100
PK1
 1 2 3 4 5 6 7 8 M 9 10 M
Ⅰ Ⅱ Ⅲ u-2 Ⅲ u-1 Ⅲ Ⅲ u-2 Ⅱ
Apico
500
400
300
200
150
100
50
Ⅰ Ⅱ Ⅲ Ⅰ Ⅰ Ⅰ Ⅰ Ⅰ Ⅰ Ⅰ
 M 1 2 3 4 5 6 7 8 M 9 10
ROP5-FspBI
500
400
300
200
150
100
 50
 M 1 2 3 5 4 6 7 8 M 9 10
 1 2 3 5 4 4 3 3 7 5/6
ROP18-Del
 M 1 2 3 4 5 6 7 8 M 9 10
 1 2 3 2 4 4 3 3 3 3
500
400
300
200
150
100
50
ROP18-UPS
 M 1 2 3 4 5 6 7 8 M 9 10
500
400
300
200
150
100
 50
 3 3 3 3 3 3
ROP5-BstUI
 1 2 3 5 4 6 7 8 M 9 10 M
500
400
300
200
150
100
50
 1 2 3 5 4 4 3 3 4 6
5'-SAG2

## Slide 2
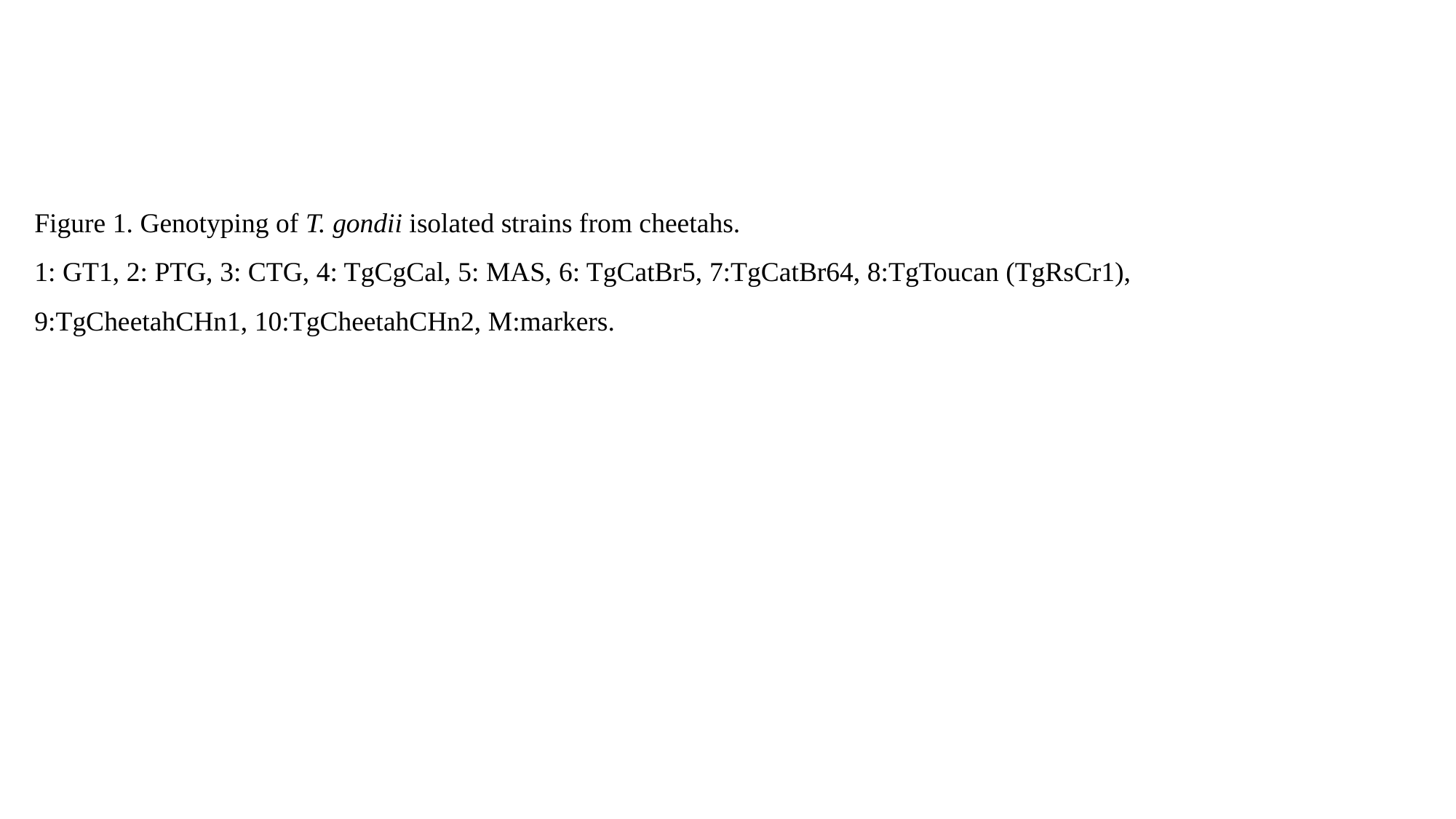

Figure 1. Genotyping of T. gondii isolated strains from cheetahs.
1: GT1, 2: PTG, 3: CTG, 4: TgCgCal, 5: MAS, 6: TgCatBr5, 7:TgCatBr64, 8:TgToucan (TgRsCr1),
9:TgCheetahCHn1, 10:TgCheetahCHn2, M:markers.
