# Supplementary material for: Direct evidence of cheetah (Acinonyx jubatus) as intermediate host of Toxoplasma gondii through isolation of viable strains
Source: BMC Vet Res. 2024 Feb 24;20:71. doi: 10.1186/s12917-024-03928-w (PMC10893619; doi:10.1186/s12917-024-03928-w)
Supplement: Supplementary file 2 — Supplementary Material 2 [file 12917_2024_3928_MOESM2_ESM.docx]

Supplementary Table 1 The survival time and antibody appearance time of mice post inoculated *Toxoplasma gondii* isolates from cheetahs (days)

|  | TgCheetahCHn1 | | TgCheetahCHn2 | |
| --- | --- | --- | --- | --- |
|  | survival time (without medicine intervention) | Tox antibody appearance time (given sulfadiazine after 2 weeks inoculation) | survival time (without medicine intervention) | Tox antibody appearance time (without medicine intervention) |
| 1 | 37 | 28 | ≥60 | 31 |
| 2 | 15 | 188 | ≥60 | 31 |
| 3 | 27 | 40 | ≥60 | 31 |
| 4 | 27 | 61 | ≥60 | 155 |
| 5 | 20 | 88 | ≥60 | 155 |
| 6 | 27 | 176 | ≥60 | 31 |
| 7 | 27 | 88 | ≥60 | 31 |
| 8 | 29 | 265 | ≥60 | 31 |
| 9 | 30 | - | ≥60 | 35 |
| 10 | 18 | - | ≥60 | 35 |
| 11 | 18 | - | - | 33 |
| 12 | 18 | - | - | 33 |
| 13 | 17 | - | - | 33 |
| 14 | 17 | - | - | 42 |
| 15 | 18 | - | - | 42 |
| 16 | 16 | - | - | - |
| 17 | 16 | - | - | - |
| 18 | 19 | - | - | - |
| 19 | 10 | - | - | - |
| 20 | 16 | - | - | - |
| 21 | 26 | - | - | - |
| 22 | 22 | - | - | - |
| 23 | 25 | - | - | - |
| Mean±SE | 21.5±1.3 | 116.8±29.6 | ≥60 | 49.9±11.1 |
